# Understanding the Predictors of Low Take-Up of the Special Supplemental Nutrition Program for Women, Infants, and Children (WIC): A Nationwide Longitudinal Study

**DOI:** 10.1007/s10995-023-03728-y

**Published:** 2023-06-07

**Authors:** Alice Guan, Akansha Batra, Hilary Seligman, Rita Hamad

**Affiliations:** 1 Department of Epidemiology & Biostatistics, University of California San Francisco (UCSF), San Francisco, CA USA; 2 Department of Medicine, UCSF, San Francisco, CA USA; 3 Center for Vulnerable Populations, UCSF, San Francisco, CA USA; 4 Department of Family & Community Medicine, UCSF, San Francisco, CA USA; 5 Philip R. Lee Institute for Health Policy Studies, UCSF, San Francisco, CA USA; 6 Department of Epidemiology and Biostatistics, University of California, 550 16th Street, 2nd Floor, San Francisco, CA 94143 USA

**Keywords:** Policy evaluation, WIC, Maternal nutrition, Child nutrition, Poverty alleviation

## Abstract

**Introduction:**

The Special Supplemental Nutrition Program for Women, Infants, and Children (WIC) is among the largest U.S. social safety net programs. Although strong evidence exists regarding the benefits of WIC, take-up (i.e., participation among eligible individuals) has steadily declined in the past decade. This study addresses gaps in our knowledge regarding predictors of WIC take-up during this time.

**Methods:**

Data were drawn from the 1998–2017 waves of the National Health Interview Study (NHIS), a serial cross-sectional study of the U.S. population. The analytic sample included 23,645 children and 10,297 women eligible for WIC based on self-reported demographic characteristics. To investigate predictors of WIC take-up, we regressed self-reported WIC receipt on a range of individual-level predictors (e.g., age, nativity, income) and state- level predictors (e.g., unemployment rate, governor’s political affiliation) using multivariable logistic regression. In secondary analyses, results were additionally stratified by race/ethnicity, time period, and age (for children).

**Results:**

For both women and children, older maternal age and higher educational attainment were associated with decreased take-up of WIC. Associations differed by race/ethnicity, time period, and state characteristics including caseload of other social programs (e.g., Medicaid).

**Discussion:**

Our study identifies groups that are less likely to take up WIC benefits for which they are eligible, thereby contributing important evidence to inform programs and policies to increase WIC participation among groups with lower take-up. As WIC evolves past the COVID-19 pandemic, special attention will be needed to ensure that resources to encourage and support the participation of racially and economically marginalized individuals are equitably distributed.

**Supplementary Information:**

The online version contains supplementary material available at 10.1007/s10995-023-03728-y.

## Significance

*What is already known on this subject?* Among women and children eligible for the Special Supplemental Nutrition Program for Women, Infants, and Children (WIC), take-up of services has been steadily declining since 2009.

*What this study adds:* In this national, longitudinal study, we found differences in take-up by individual-level characteristics (e.g., maternal age and educational attainment) and state-level characteristics (e.g., caseload of other social programs). Given the increased needs for social support programs following the COVID-19 pandemic and our findings of disparate take-up of WIC benefits, special attention is needed to ensure that program accessibility is equitable.

**Supplementary Information:**

The online version contains supplementary material available at 10.1007/s10995-023-03728-y.

## Introduction

The quality of perinatal and early childhood nutrition has consequences for lifelong chronic disease risk among women and children (Mameli et al., 2016). Socioeconomic and racial disparities in dietary quality also contribute to health disparities across the lifespan. For instance, higher socioeconomic status has been associated with having healthier food options (Kell et al., 2015), and low-income Black and Hispanic women have been found to have less healthy diets with respect to national nutrition recommendations (Kirkpatrick et al., 2012; Satia, 2009). Policies and programs that improve dietary quality among racially and economically marginalized subgroups may hold promise for improving population-level health disparities (Seligman & Hamad, 2021).

The Special Supplemental Nutrition Program for Women, Infants, and Children (WIC) is one of the largest U.S. safety net programs, and was established in the early 1970s to improve the health of low-income families. WIC provides food benefits, health education, and screenings/referrals for low-income pregnant and postpartum women and infants and children up to age 5. WIC serves approximately half of all infants, one fourth of all pregnant/postpartum women and one fourth of children aged 1–4 in the United States (Oliveira, 2009; US Department of Agriculture, Food and Nutrition Service, 2020). Participation in WIC is associated with positive health outcomes, and recent revisions to aligning the nutrition package with federal guidelines (e.g., adding a fruit and vegetable benefit, requiring healthier options) have further improved maternal and child nutrition and health (Guan et al., 2021; Hamad et al., 2019a, 2019b, 2019c; Hamad et al., 2019a, 2019b, 2019c). WIC participation may improve birth outcomes, including birth weight, gestational age, and infant mortality (Fingar et al., 2017; Venkataramani et al., 2022). Prenatal and early childhood participation in WIC can also result in long-lasting benefits to child health including improved academic and mental health outcomes (Chorniy et al., 2020; Jackson, 2015). WIC may also reduce disparities; a recent study revealed that WIC participation was associated with improved health of Black and Hispanic infants and a reduction in racial/ethnic disparities in birth outcomes compared to White infants (Testa & Jackson, 2021).

Despite the potential benefits of WIC participation, historical challenges have resulted in a significant number of eligible individuals not participating. In fact, this pernicious issue has led to a 30% decline in WIC-eligible individuals receiving the benefits for which they qualify between 2010 and 2019 (Tiehen, 2020). There are numerous reasons why WIC-eligible individuals do not participate, including: the extensive and time-consuming application process (Beckmann et al., 2000; Rosenberg et al., 2003), unawareness of eligibility (S. Gray et al., 1995; Rush et al., 1988), and experiences or perceptions of stigma (Chauvenet et al., 2019). Furthermore, it is important to recognize that under-enrollment in the WIC program is rarely caused by a single factor, but rather by complex relationships of individual, interpersonal, and institutional-level factors (Morrissey, 2010, 2016). Participation among WIC-eligible individuals also varies by state. For instance, in 2017, 64% of eligible individuals in Maryland participated compared to 36% in Montana (U.S. Department of Agriculture, 2020). Given the positive health benefits associated with WIC participation, reduced enrollment could limit its population-level impact, particularly among racially and economically marginalized families.

Potential determinants of WIC take-up remain understudied. On the individual level, several studies conducted in limited geographies [e.g., New York City (Liu & Liu, 2016) and San Francisco (Faed et al., 2014)] have found that, for women, predictors of participation included access to social support, transportation, and health insurance. A larger number of studies have investigated predictors of take-up for children (Whaley et al., 2020; Woelfel et al., 2004). For instance, one study using national panel data from the Survey of Income and Program Participation found greater participation among Hispanic children (vs. non-Hispanic White) and those with less educated parents (vs. more highly educated) (Jackson & Mayne, 2016). However, much of this previous research has focused on identifying individual-level barriers to participation rather than seeking to identify groups which underutilize WIC. State-level characteristics associated with WIC participation are even less studied, although one study using state-level data from 1983 to 2006 found that take-up was associated with caseload of state social programs (e.g., Medicaid, other nutrition assistance programs) and unemployment rates (Swann, 2010). There is limited evidence examining multilevel predictors of WIC participation for both women and children using a nationwide sample, particularly using more recent data that can illuminate changes related to the shifting U.S. political landscape. In this study, we sought to fill this critical gap in the literature by investigating individual- and state-level predictors of WIC take-up among eligible women and children using rich national longitudinal data. A better understanding of the predictors of WIC take-up (i.e., participation among eligible individuals) will enable governments, community groups, and clinicians to better design and target policies and interventions to enhance WIC participation.

## Methods

### Data

Data were compiled from the 1998–2017 waves of the National Health Interview Survey (NHIS), a serial cross-sectional annual household survey representative of the U.S. population (N = 1,989,149) (*National Center for Health Statistics (NCHS); 1997–2018 NHIS; Data, Questionnaires and Related Documentation*, 2020). WIC eligibility is set annually by the federal government using criteria including income below 185% of the federal poverty line (which adjusts for household size), currently pregnant or postpartum for women, and age less than 5 years for children. Thus, the analytic sample of children was restricted to those under the age of 5 (N = 143,307). For women, NHIS did not consistently include questions on whether they were currently or recently pregnant or breastfeeding. However, having an infant could indicate that women themselves were recently pregnant. Therefore, we restricted the analytic sample for women to those with children under the age of 1 (N = 172,954) to capture likely postpartum status. We additionally restricted both samples using an indicator of WIC eligibility, which was imputed (i.e., presumed) from federal criteria based on participant’s self-reported annual household income and family size based on state of residence (as eligible income cutoffs varied at the state level) and year. While imputing eligibility for WIC and other safety net programs using self-reported demographics is imperfect due to errors in self-reported data, it is a standard technique in the literature (Collin et al., 2023; Shields-Zeeman et al., 2021), as linking administrative datasets is often not possible. The final analytic samples included 23,645 children and 10,297 women (eFigure 1).

### Measures

The primary outcome of interest was a dichotomous measure of participant-reported WIC receipt, which NHIS collects for each individual in the household. Specifically, participants were asked, “At any time during [the last calendar year] did [you/any family members living here] receive benefits from the WIC program, that is, the Women, Infants, and Children program?” Individual-level predictors included: parent (or woman’s own) age (5 categories ranging from under 25 to 40 or older), marital status (vs. not), educational attainment (less than high school, high school, some college or more), employment status (employed in the past week vs. not); child age (5 categories for each year of eligibility, 0 to 4); family size; inflation-adjusted household income; nativity (any immigrant in household vs. none); census region of residence (Northeast, Midwest, South, West); and race/ethnicity. The latter was categorized into non-Hispanic White, non-Hispanic Black, Hispanic, and other, since other subgroups contained fewer observations that might result in unstable estimates. This variable was constructed based on two questions in the NHIS, which separately assessed Hispanic origin and race. Because the generosity of state programs such as Medicaid may be a good indicator of how well the state supports other safety programs like WIC, we included several state-level variables in this study. State-level characteristics were drawn from online governmental databases and the University of Kentucky Poverty Research Center (Bureau of Economic Analysis, 2021; Bureau of Labor Statistics, 2021; University of Kentucky Center for Poverty Research, 2019), and included mean gross domestic product (GDP) per capita, unemployment rate, political affiliation of the governor (Democrat vs. not), state earned income tax credit (EITC) rate, and state caseload of the Temporary Assistance for Needy Families program (TANF), Supplemental Nutrition Assistance Program (SNAP), and Medicaid. Caseload of social programs were population-standardized ([caseload/population] × 100). State-level characteristics were merged with NHIS individual-level data based on state of residence and interview year.

### Statistical Analysis

We first tabulated descriptive statistics for WIC-eligible women and children. We used multivariable logistic regression to investigate the association of a range of individual- and state-level predictors of self-reported WIC participation. We estimated odds ratios and 95% confidence intervals, and considered p-values of < 0.05 to be statistically significant. Several secondary analyses were conducted. First, we stratified analyses by race/ethnicity, since factors related to structural racism, immigration status, and language might result in differences for each subgroup. Second, participation in social programs may reflect national economic conditions (e.g., poverty) (Carlson et al., 2017). Thus, to examine whether the predictors of WIC take-up differed over time in response to programmatic changes and economic and political conditions, associations were examined separately for different time periods (pre-Great Recession [1997–2008], post-Great Recession [2009–2017]). This analysis was also intended to capture potential differences in predictors before and after 2009, when WIC participation began to decline. Finally, for children, we also evaluated associations separately for infants and children, since increased administrative tasks are needed to continue participation past age one (Bitler et al., 2003; Geller et al., 2012; Whaley et al., 2020).

### Ethical Approval

Ethical approval for this study was provided by the institutional review board at the senior author’s university (protocol #17-23255).

## Results

### Sample Characteristics

Approximately half of likely WIC-eligible children (44.3%) and women (39.1%) reported receiving WIC (Table 1). A majority of the child sample was Hispanic (35.7%), with mothers under age 25 (36.1%), with at least one parent who was foreign born (76%). For women, 36.4% were under 25, 39% were immigrants, 46.6% were married, and 40.8% were employed in the past week. The largest proportion of both children and women samples resided in the Southern census region (40.4% and 40.9%).

**Table 1:** Sample Characteristics

	Children		Women	
	Percent	Mean (SD)	Percent	Mean (SD)
Reported WIC receipt	44.3		39.1	
Woman’s age				
Under 25	36.1		36.4	
25–29	29.4		30.2	
30–34	18.8		20.5	
35–39	10.3		9.8	
40 or older	5.3		3.0	
Child age				
Infant	19.7			
1 year old	21.4			
2 years old	20.3			
3 years old	19.6			
4 years old	19.0			
Parental education				
Less than high school	32.7		33.2	
High school	31.5		31.8	
Some college or more	35.8		35.0	
Race/ethnicity				
White	30.5		33.3	
Black	21.1		24.1	
Hispanic	35.7		32.5	
Other	12.7		10.0	
Immigrant household^a^	76.0		39.0	
Family income (thousands of USD)		24.7 (14.0)		22.9 (14.4)
Parent employed in past week	40.7		40.8	
Parent married	53.4		46.6	
Family size		4.0 (1.5)		4.0 (1.6)
State GDP (thousands of USD)		48.9 (10.8)		49.2 (10.9)
State unemployment rate		6.1 (2.1)		6.1 (2.1)
State SNAP caseload		4.7 (2.2)		4.8 (2.2)
State TANF caseload		0.7 (0.5)		0.7 (0.5)
State Medicaid caseload		16.6 (5.4)		16.7 (5.4)
State has Democrat governor	38.0		37.3	
State EITC rate (%)		4.5 (10.3)		4.6 (10.1)
Census region				
Northeast	12.5		11.9	
Midwest	18.3		19.3	
South	40.4		40.9	
West	28.8		27.9	

Sample includes WIC-eligible women with children under age 1 (N = 10,297), and children under the age of 5 (N = 23,645) who participated in the National Health Interview Survey during 1998–2017. The samples were additionally restricted based on imputed WIC eligibility, defined using household income, family size, and state income eligibility criteria for WIC

*WIC* Special supplemental nutrition program for women, Infants and Children; *USD* U.S. dollars; *GDP* Gross domestic project; *SNAP* Supplemental nutritional assistance program; *TANF* Temporary assistance for needy families; *EITC* Earned income tax credit. Caseload variables above (for SNAP, TANF and Medicaid) represent total caseload in the population (i.e., caseload/population*100)

^a^In child sample, immigrant household was defined as having at least one immigrant parent; in women sample, immigrant household defined based on self-reported nativity

### Predictors of WIC Take-Up

**Fig. 1:**
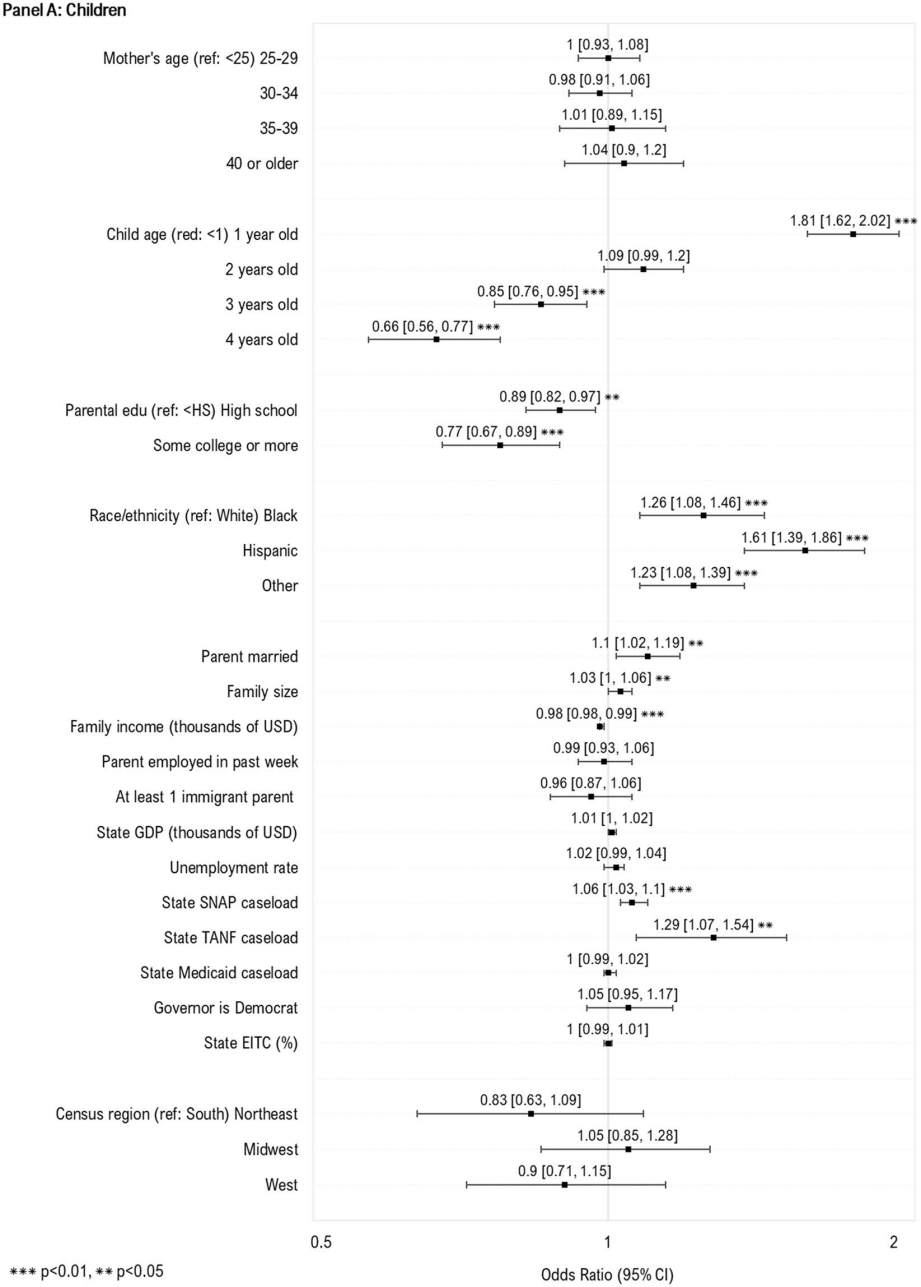

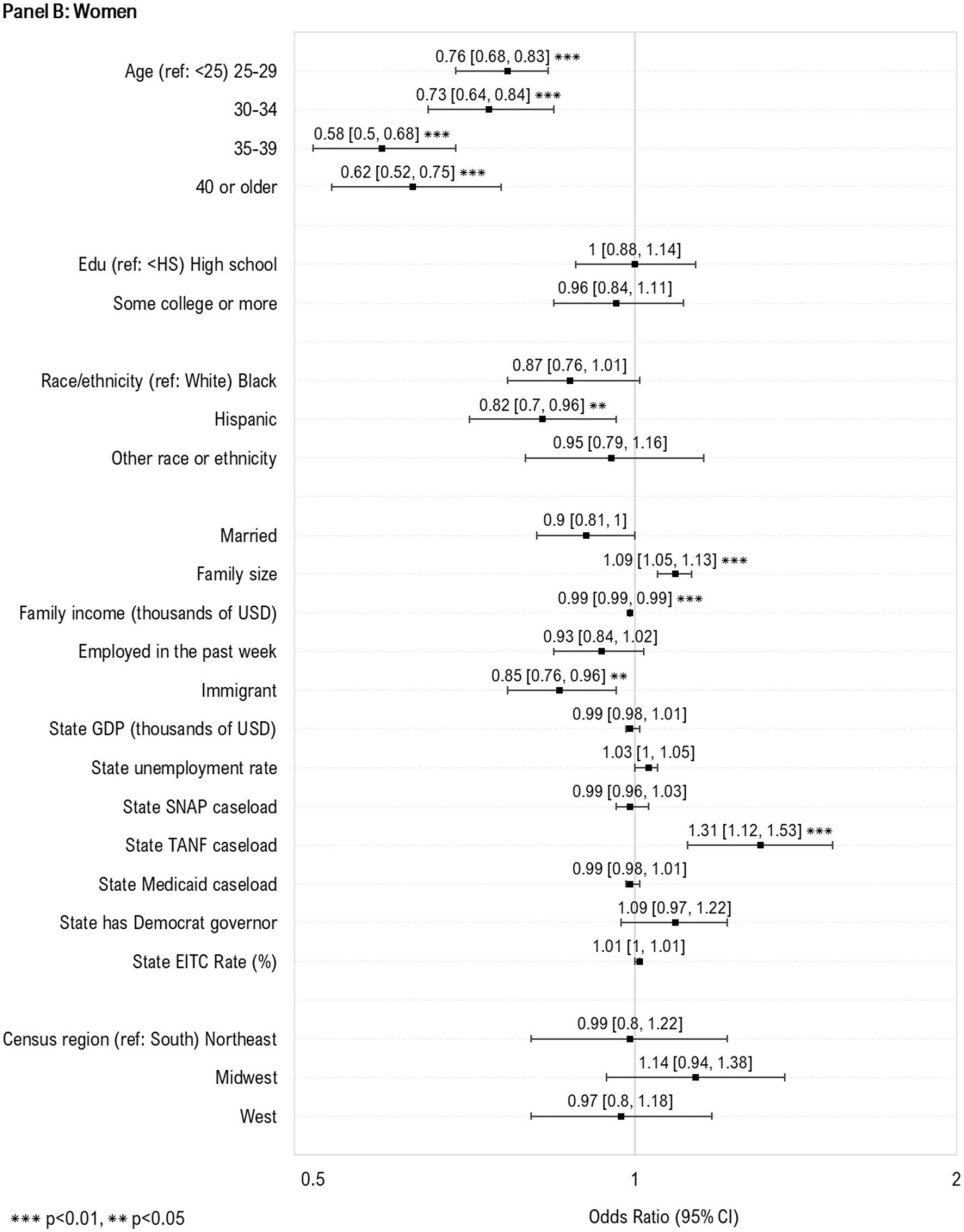
Individual- and state-level predictors of WIC take-up among women and children likely eligible for WIC. Sample includes WIC eligible women with children under age 1 (N = 10,297) and children under the age of 5 (N = 23,645) who participated in the National Health Interview Survey during 1998–2017. The samples were additionally restricted based on imputed WIC eligibility, defined using household income, family size, and state income eligibility criteria for WIC. Estimates in the above plot represent odds ratios from multivariable logistic regression models. To preserve symmetry and ease interpretation, results are plotted on the natural log scale. *WIC* Special supplemental nutrition program for women, infants and children; *Edu* education; *USD* U.S. dollars; *GDP* Gross domestic project; *SNAP* Supplemental nutritional assistance program; *TANF* Temporary assistance for needy families; *EITC* Earned income tax credit. Caseload variables above (for SNAP, TANF and Medicaid) represent total caseload in the population (i.e., caseload/population*100)

We next examined individual- and state-level predictors of WIC take-up (Fig. 1). For children, we observed increased WIC participation among those with non-White race or ethnicity (compared to White), 1-year-olds (compared to infants), and those with lower parental education, lower family income, those with married parents, larger families, and higher state caseload of SNAP and TANF. For women, we observed increased take-up of WIC among those who were younger, Hispanic, US-born, had lower family income, had larger families, and higher state caseloads of TANF. No additional factors were associated with increased take-up.

### Predictors of WIC Take-Up by Race/Ethnicity

**Fig. 2:**
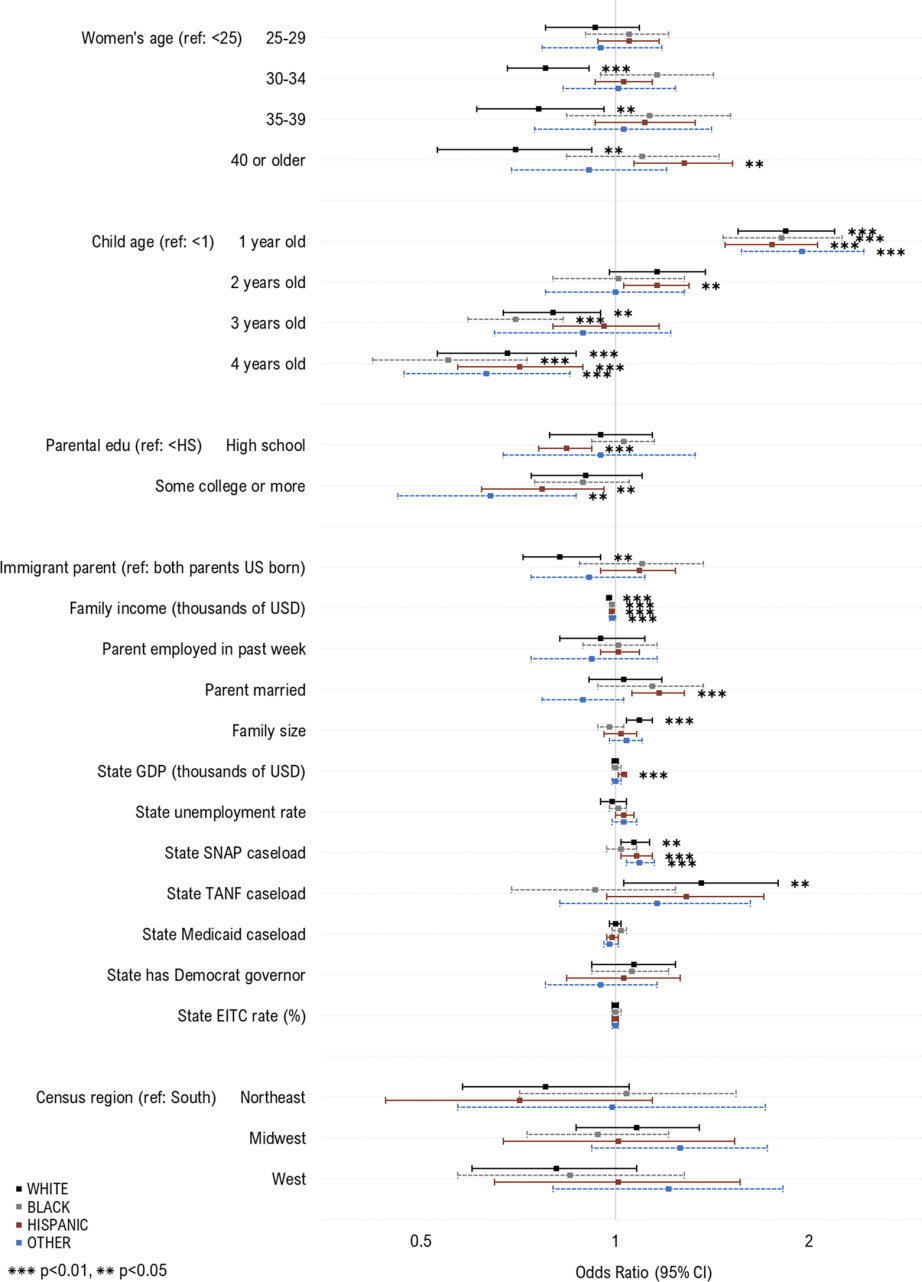

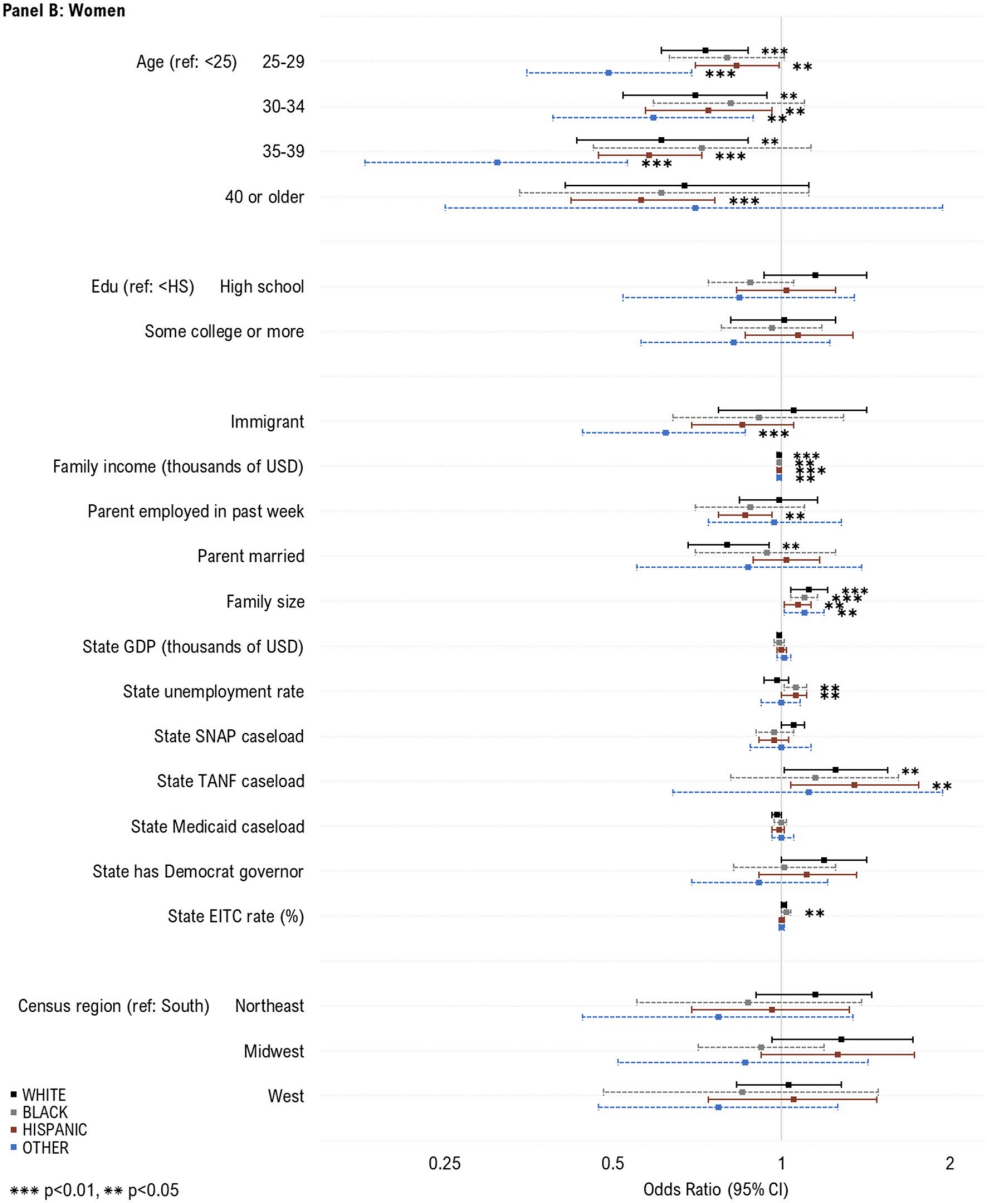
Individual- and state-level predictors of WIC take-up among children and women likely eligible for WIC, by race/ethnicity. Sample includes WIC eligible women with children under age 1 (White, N = 2893; Black, N = 2056; Hispanic, N = 2802; Other race, N = 819) and children under the age of 5 (White, N = 5470; Black, N = 3622; Hispanic, N = 6613; Other race, N = 2226) who participated in the National Health Interview Survey during 1998–2017. The samples were additionally restricted based on imputed WIC eligibility, defined using household income, family size, and state income eligibility criteria for WIC. Estimates in the above plot represent odds ratios from multivariable logistic regression models. To preserve symmetry and ease interpretation, results are plotted on the natural log scale. *WIC* Special supplemental nutrition program for women, infants and children; *Edu* education; *USD* U.S. dollars; *GDP* Gross domestic project; *SNAP* Supplemental nutritional assistance program; *TANF* Temporary assistance for needy families; *EITC* Earned income tax credit. Caseload variables above (for SNAP, TANF and Medicaid) represent total caseload in the population (i.e., caseload/population*100) (Color figure online)

We then examined predictors of WIC take-up stratified by race/ethnicity (Fig. 2). For children across all racial and ethnic groups, we observed increased take-up among 1-year-olds, and those with lower family income. For White children, increased take-up was additionally associated with younger maternal age, larger family size and higher state caseload of SNAP and TANF, and having both US born parents. For Hispanic children, increased take-up was also observed among those with older mothers, married parents, lower parental education, andhigher state GDP and caseload of SNAP. For children of other racial/ethnic groups, increased take-up was associated with lower parental education and higher state caseload of SNAP.

For women of all racial/ethnic groups, increased take-up of WIC was associated with younger age, lower family income, and larger family size. For White women, increased take-up was additionally associated with being unmarried, and having higher state caseload of TANF. For Black women, increased take-up was additionally associated with state unemployment and EITC rate. For Hispanic women, increased take-up was associated with being unemployed, and higher state unemployment rate and caseload of TANF. For women of other racial/ethnic groups, being US-born was additionally associated with increased take-up.

No additional factors were associated with increased take-up in race/ethnicity stratified models.

### Predictors of WIC Take-Up Over Time

**Fig. 3:**
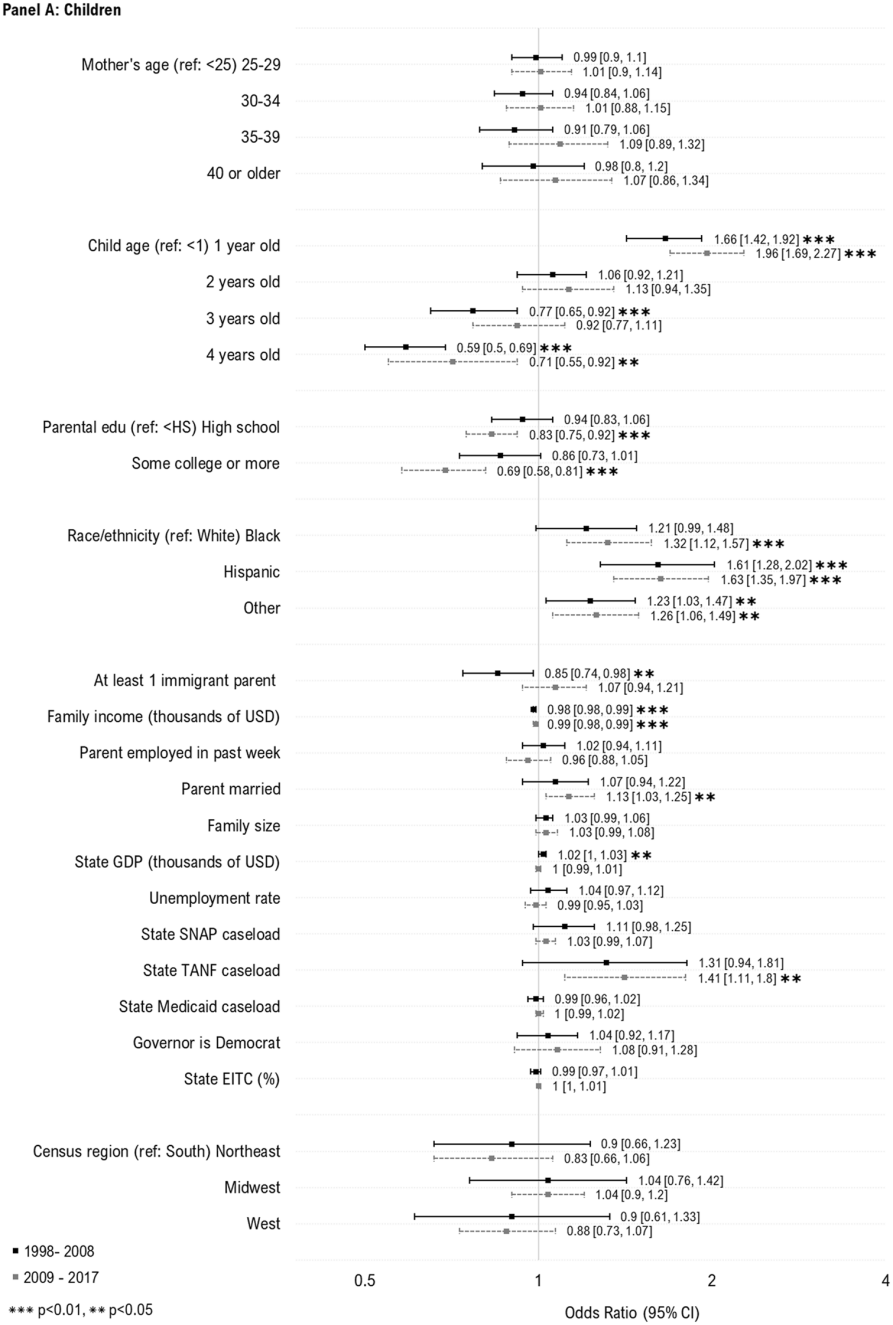

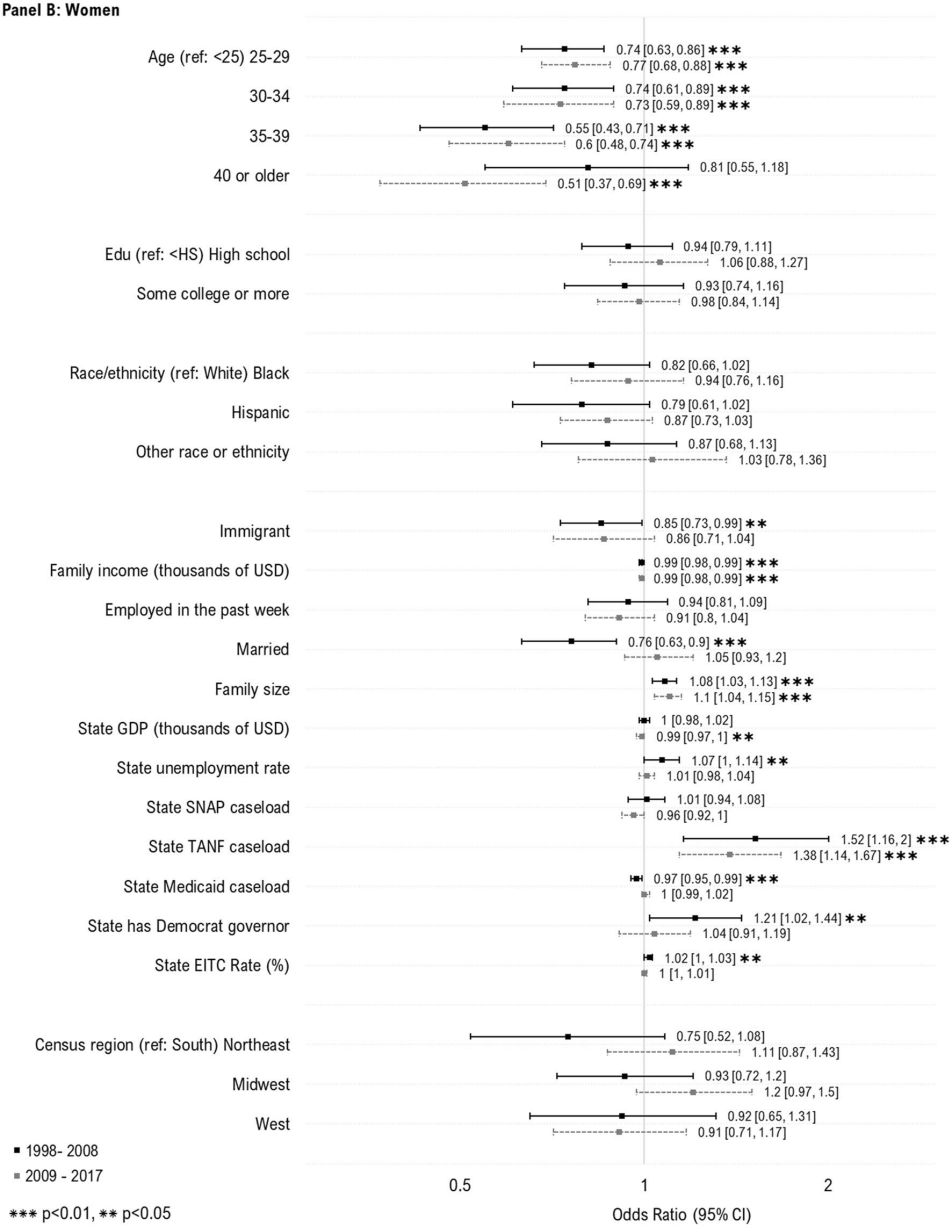
Individual- and state-level predictors of WIC take-up among women and children likely eligible for WIC, by time period. Sample includes WIC eligible women with children under age 1 (1998–2008, N = 4033; 2009–2017, N = 4537) and children under the age of 5 (1998–2008, N = 8437; 2009–2017, N = 9494) who participated in the National Health Interview Survey during 1998–2017. The samples were additionally restricted based on imputed WIC eligibility, defined using household income, family size, and state income eligibility criteria for WIC. Estimates in the above plot represent odds ratios from multivariable logistic regression models. Labels in the figure depict “Odds ratio [95% Confidence interval]”. To preserve symmetry and ease interpretation, results are plotted on the natural log scale. *WIC* Special Supplemental Nutrition Program for Women, Infants and Children; *Edu* education; *USD* U.S. dollars; *GDP* Gross domestic project; *SNAP* Supplemental Nutritional Assistance Program; *TANF* Temporary Assistance for Needy Families; *EITC* Earned income tax credit. Caseload variables above (for SNAP, TANF and Medicaid) represent total caseload in the population (i.e., caseload/population*100)

Next, we evaluated predictors of take-up before and after 2009, corresponding with both the Great Recession and when WIC participation began declining (Fig. 3). For children across both time-periods, increased take-up was observed among 1-year-olds, children who were Hispanic or other race, and those with lower family incomes. In the period before 2009, increased take-up was observed among infants, those with US-born parents, and higher state GDP. After 2009, increased take-up was observed among those with lower parental education, married parents, higher state caseload of TANF.

For women across time periods, increased take-up was associated with younger age and lower family income, smaller families, and lower state TANF caseload. Prior to 2009, increased take-up was additionally associated with being US-born, unmarried, having lower state Medicaid caseload, higher state unemployment and EITC rate, and living in a state with a democratic governor. After 2009, increased take-up was observed among states with lower GDP.

No additional factors were associated with increased take-up.

### Predictors of WIC Take-Up by Child Age

**Fig. 4:**
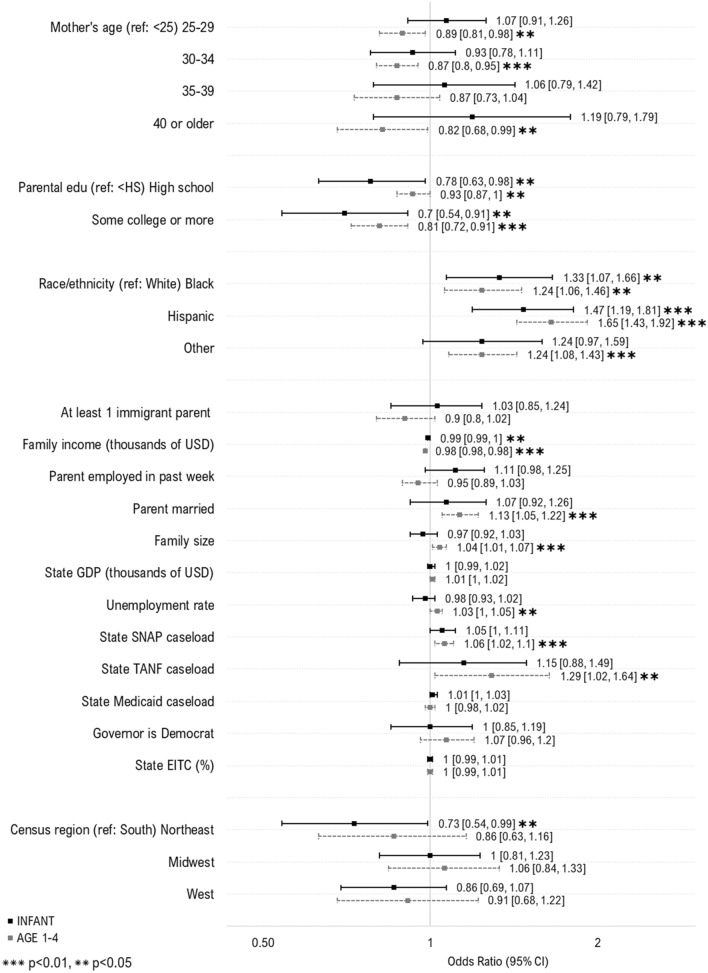
Individual- and state-level predictors of WIC take-up among children likely eligible for WIC, by age. Sample includes WIC-eligible children (Infants, N = 3595; Children aged 1–4, N = 14,336) who participated in the National Health Interview Survey during 1998–2017. The samples were additionally restricted based on imputed WIC eligibility, defined using household income, family size, and state income eligibility criteria for WIC. Estimates in the above plot represent odds ratios from multivariable logistic regression models. Labels in the figure depict “Odds ratio [95% Confidence interval]”. To preserve symmetry and ease interpretation, results are plotted on the natural log scale. *WIC* Special Supplemental Nutrition Program for Women, Infants and Children; *Edu* education; *USD* U.S. dollars; *GDP* Gross domestic project; *SNAP* Supplemental Nutritional Assistance Program; *TANF* Temporary Assistance for Needy Families; *EITC* Earned income tax credit. Caseload variables above (for SNAP, TANF and Medicaid) represent total caseload in the population (i.e., caseload/population*100)

Finally, we evaluated predictors of WIC take-up stratified by children’s age (Fig. 4). For all children, increased take-up was observed for those with lower parental education and family income, and those who were Black or Hispanic. For children aged 1–4, increased take-up was observed among those with married parents, larger families, and in states with higher unemployment rates and caseloads of SNAP and TANF. No additional factors were associated with increased take-up.

## Discussion

In this analysis of a national longitudinal sample of likely WIC-eligible individuals, several factors were consistently associated with WIC take-up. Across racial/ethnic groups and over time, increasing age was associated with decreased take-up of WIC among women. This finding is consistent with national reports that prenatal WIC receipt is highest among women under age 20 (Driscoll & Osterman, 2018). Additionally, parenthood at a younger age has been found to be more prevalent among socioeconomically disadvantaged groups (Berzin & De Marco, 2010; Penman-Aguilar et al., 2013), which suggests that young mothers who may be more in need of assistance are being reached by WIC. For children we observed decreased take-up of WIC among older children (e.g., age 3- and 4) and increased take-up among 1-year-olds compared with infants. This is somewhat inconsistent with prior evidence that participation was highest among infants (Gray et al., 2021; US Department of Agriculture Food & Nutrition Services, 2006), and reports of up to 23% attrition of WIC infants once they turn one (Jacknowitz & Tiehen, 2010). One potential explanation for this finding could be that WIC supports postpartum women and their infants differentially based on whether or not a mother is breastfeeding (i.e., WIC food packages are offered to women—rather than the infant—if they are exclusively breastfeeding, up to 6 months postpartum). Therefore, it is possible that differences we observed could be due to misclassification of WIC receipt. Our findings also provide insights into factors that influence take-up differently among infants versus older children. For example, higher state caseload of TANF was associated with increased take-up among children aged 1–4, suggesting that states which provide more support do a better job of retaining WIC-eligible children beyond infancy. However, we did not generally observe an association between state EITC rate and WIC take-up, which may be due to the fact that EITC benefits are administered through the tax system, which reflects a fundamentally different process than the application process for WIC.

In terms of differences by race/ethnicity, we found increased participation among Hispanic and Black children. This is consistent with USDA reports, which found coverage rates to be highest among Hispanic/Latino children (Gray et al., 2021). In contrast, we observed lower participation among Hispanic women. Our study extends other descriptive analyses and provides evidence that while Hispanic children are being substantially reached by the WIC program, eligible Black and Hispanic women are continuing to miss out and should thus be targeted through outreach. Tailored messaging about WIC may be needed for groups that are less likely to take up WIC services, for instance through text messaging campaigns, social media and digital marketing strategies, and digital referral interventions, which have been found to be promising approaches for increasing WIC participation (Paige et al., 2023). Additionally, we found that nativity was a barrier for participation among women of other racial/ethnic groups. Further research using an intersectional lens is needed to understand how race/ethnicity and immigration interact to affect participation in social programs.

We found that different socioeconomic factors played a role in WIC take-up. Specifically, higher income was associated with lower take-up, which is consistent with previous studies (Whaley et al., 2020). Additionally, we observed lower take-up in WIC among children with more educated parents. Prior studies have found that women who did not complete high school were more likely to participate in WIC than college graduates (Bitler & Currie, 2005). Additionally, the psychological cost (i.e., stigma) of participating in social programs has been found to increase with educational attainment (Manchester & Mumford, 2009), which could explain the gradient we observed. Our findings regarding the overall null association of employment with WIC take-up are somewhat inconsistent with previous studies, one of which found that employment may serve as a barrier to WIC receipt (e.g., not being able to take time off work to attend WIC appointments) (Liu & Liu, 2016). However, this is likely due to differences in measurement of employment.

While WIC is a federal program that is intended to be uniform nationwide, we found differences by state characteristics even after adjusting for individual factors, implying that state context may differentially affect participation. For example, for both women and children, higher state caseload of TANF was associated with increased take-up of WIC, and we observed differences in the magnitude of this association before and after 2009. One potential mechanism through which state characteristics operate could be differences in the ways that social programs are generally overseen. Administrative burdens are often intentionally designed to limit access to services, and often disproportionately impact groups which are already marginalized by structural racism and economic factors (Herd & Moynihan, 2018). For instance, it is possible that states with greater caseloads of other social programs (e.g., TANF) impose less transactional and administrative costs for applying or have more WIC clinics available (Bitler et al., 2003). One such process of reducing administrative burden at the state level involves simplifying the eligibility process by automatically considering individuals enrolled in other social programs, such as Medicaid or SNAP, to be “adjunctively eligible” for WIC (*7 CFR § 246.7—Certification of Participants.*, 1985). A recent report found that approximately half of state WIC agencies do not currently have agreements between these social programs (Neuberger, 2021), pointing to a potential policy intervention to facilitate and increase enrollment.

There are several limitations to this study. First, we imputed eligibility based on self-reported demographics, so the denominator may include individuals who were not eligible. Specifically, because NHIS did not consistently measure pregnancy status, we opted to restrict the women sample to those with children under 1. This does not capture women in the prenatal period, but only the postpartum period. Additionally, although breastfeeding status impacts a woman’s eligibility for WIC, this information was not consistently collected by NHIS. Not accounting for breastfeeding may have resulted in measurement error in defining out sample of eligible women. Furthermore, the exposure, outcomes, and covariates were self-reported and therefore may suffer from measurement error and standard reporting biases. In particular, prior studies have shown that people may not accurately report safety net program participation (Meyer & Mittag, 2019; Meyer et al., 2018). Errors in self-reported participation have also been found to differ by race/ethnicity and household characteristics (Celhay et al., 2021). Unfortunately, administrative data from WIC are difficult to access and not easily linked with rich demographic variables like those from NHIS, but these linkages can be considered in future research. Additionally, NHIS did not collect information on several measures potentially relevant for WIC take-up, including stigma, perceptions of government, or access issues. Thus, we may be identifying predictors that are correlated with these unmeasured characteristics. Furthermore, the way that we assessed the caseload of various state social programs (e.g., TANF, SNAP) may not reflect the differences in needs based on underlying state demographics. Finally, using state-level predictors potentially masks heterogeneity within states (e.g., by county). For instance, it is likely that there are additional differences in the administration of WIC services at geographically local, sub-state levels, as well as differences in infrastructure or resources which could impact service delivery at this level.

Our study contributes important evidence that could inform programs and policies to increase WIC participation among groups with lower take-up. As WIC evolves past the COVID-19 pandemic, attention is needed to ensure that resources to support the participation of racially and economically marginalized individuals are equitably distributed. Few studies have examined interventions to increase take-up of WIC, although one recent study found that changing WIC from paper vouchers to electronic debit cards increased take-up (Vasan et al., 2021). Amidst the COVID-19 pandemic, WIC clinics have begun to allow health departments to provide electronic certification, which have increased participation in Kentucky and California (Dearinger, 2020; Whaley & Anderson, 2021). Additionally, prior work has found that the Great Recession led to enrollment of different individuals in safety net programs as a result of the widespread economic crisis (Hamad et al., 2019a, 2019b, 2019c). All this suggests that flexible service delivery and expansion of technological advances in remote support may be promising avenues for WIC to address the long-term economic backlash of the pandemic including among newly served populations, and our study will help to inform such interventions.

### Supplementary Information

Below is the link to the electronic supplementary material.

Supplementary file1 (DOCX 39 KB)

## Data Availability

Data described in the manuscript, codebook, and analytic code will not be made available because analysis was conducted using restricted geographic data, which could lead to the identification of participants. Interested investigators can apply for these data at https://www.cdc.gov/rdc. Detailed documentation can be obtained by contacting RDCA@cdc.gov.
