# Supplementary material for: Understanding the Predictors of Low Take-Up of the Special Supplemental Nutrition Program for Women, Infants, and Children (WIC): A Nationwide Longitudinal Study
Source: Matern Child Health J. 2023 Jun 7;27(10):1795–810. doi: 10.1007/s10995-023-03728-y (PMC10247269; doi:10.1007/s10995-023-03728-y)
Supplement: Supplementary file 1 — Supplementary file1 (DOCX 39 KB) [file 10995_2023_3728_MOESM1_ESM.docx]

**SUPPLEMENTAL MATERIALS**

**eFigure 1.** Sample flowchart

**eFigure 1. Sample flowchart**

Footnote: WIC eligibility was imputed using federal criteria. This was determined using self-reported household income and family size, and state income eligibility criteria for the WIC. **Abbreviations**: WIC: Special Supplemental Nutrition Program for Women, Infants and Children
